# Reaction of Electrons with DNA: Radiation Damage to Radiosensitization

**DOI:** 10.3390/ijms20163998

**Published:** 2019-08-16

**Authors:** Anil Kumar, David Becker, Amitava Adhikary, Michael D. Sevilla

**Affiliations:** Department of Chemistry, Oakland University, Rochester, MI 48309, USA

**Keywords:** quasi-free electron, solvated electron, low energy electrons, ion-beam radiation, radiosensitization

## Abstract

This review article provides a concise overview of electron involvement in DNA radiation damage. The review begins with the various states of radiation-produced electrons: Secondary electrons (SE), low energy electrons (LEE), electrons at near zero kinetic energy in water (quasi-free electrons, (e^−^_qf_)) electrons in the process of solvation in water (presolvated electrons, e^−^_pre_), and fully solvated electrons (e^−^_aq_). A current summary of the structure of e^−^_aq_, and its reactions with DNA-model systems is presented. Theoretical works on reduction potentials of DNA-bases were found to be in agreement with experiments. This review points out the proposed role of LEE-induced frank DNA-strand breaks in ion-beam irradiated DNA. The final section presents radiation-produced electron-mediated site-specific formation of oxidative neutral aminyl radicals from azidonucleosides and the evidence of radiosensitization provided by these aminyl radicals in azidonucleoside-incorporated breast cancer cells.

## 1. Introduction

Free electrons are produced in all ionizing radiation processes and their radiation chemistry depends on their energy, state of solvation and the nature of proximate reactive species. In this review, we provide an overview of these processes starting from the formation of secondary electrons (SE), then to low energy electrons (LEE) on to electrons at near zero kinetic energy in water (quasi-free electrons, (e^−^_qf_)), electrons in the process of solvation in water (presolvated electrons, e^−^_pre_) and to the fully solvated electron (e^−^_aq_). This overview points out that each of these species exhibit different reactivities with biomolecules, including product formation. In Section 2, we begin with an overview to characterize these species and their relative energies. Subsequently, we present our latest computational chemistry results on the nature of the fully solvated electron, which 60 years after its discovery is still a source of some controversy. Recent results suggest that a simple tetrahedral “cavity model” of the solvated electron fully predicts its properties and reactivity with DNA-bases (Section 3). The current status of experimental and theoretical works on the reduction potentials of DNA-anion radicals formed by addition of solvated electrons with DNA-models are also described. In Section 4, the role of electrons and excitations are pointed out as an important pathway in ion-beam irradiations of biomolecules. Finally, in Section 5, we show that selective azidonucleosides which capture radiation-produced electrons are showing promise in the augmentation of radiation damage in cancer cell lines.

## 2. Various States of Radiation-Produced Electrons

### 2.1. Introduction

The interaction of high energy radiation (UV, X-ray, γ-ray, ion-beam) with liquid water, transiently generates excited states (H_2_O*), H_2_O^•+^, and secondary electrons (SE) [1,2,3,4,5,6,7,8]. On a femtosecond time scale, H_2_O* autoionizes to form H_2_O^•+,^ which then deprotonates to form OH^•^ and H_3_O^+^, or dissociates to OH^•^ and H^•^ [2,9]. However, SE lose energy to form, in sequence, low energy electrons (LEE), quasi-free electrons (e^−^_qf_), then prehydrated electrons (e^−^_pre_), which are rapidly solvated by the surrounding water molecules within a picosecond forming the “solvated electron” (e^−^_aq_) [10,11,12].

**Scheme 1:**
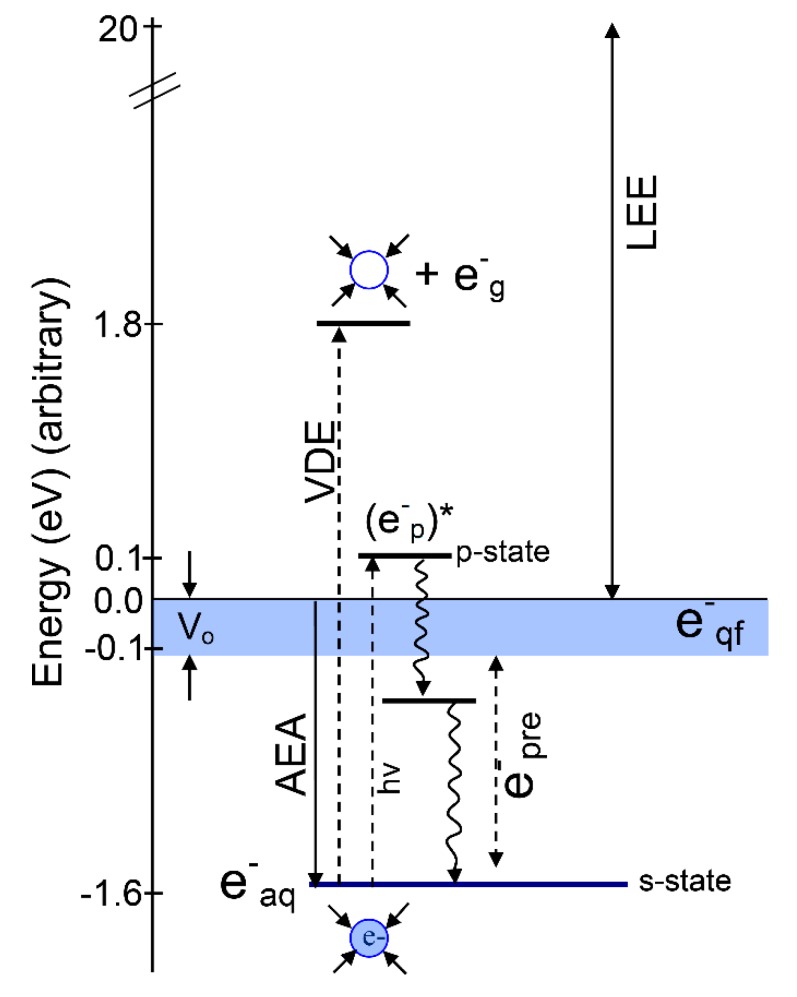
Energy diagram showing the addition of an electron to bulk water. The various energy values were taken from experiment. Adiabatic electron affinity (AEA, 1.6 eV) equals the negative of the free energy of solvation [32,33]. The vertical detachment energy (VDE) = 3.4 eV. *V*_o_ is taken from the experiment by Coe et al. [25]. The difference between VDE and AEA gives the relaxation energy (1.8 eV). The vertical excitation energy s→p* (1.73 eV) is taken from [34,35]. The fast solvent relaxation around (e^−^_p_)* within 200 fs before internal conversion is shown as e^−^_pre_ which lies below the conduction band (blue band). e^−^_pre_ lies in the energy range 0 eV to −1.6 eV which is between e^−^_qf_ and e^−^_aq (_blue circle). The excited state energy levels of e^−^_aq_ are based on those in reference [10]. Note that the zero of energy has the electron at zero eV and equilibrated (relaxed) water; e^−^_g_ refers to a zero-eV gas phase electron. LEE are low energy electrons.

Reactions of radiation-produced electrons with DNA-models have been extensively studied using pulse radiolysis, electron spin resonance (ESR), and theory [3,5,6,7,13,14,15,16,17]. The reactivity of radiation-produced electrons is very dependent on the medium and their energies and these are defined as: (i) Electrons with kinetic energy between 0 and 20 eV are termed low-energy electrons (LEE) [1,2,3,4,5,6,7,8] and are produced in large numbers (~4 × 10^4^ electrons per MeV energy deposited) along the tracks of the ionizing radiation [11]. These LEEs > 10 eV can cause ionizations and at 10 eV and below are shown to cause single and double-strand breaks in plasmid DNA through a dissociative electron attachment (DEA) mechanism [8,18]. Collisions of LEEs with the aqueous medium generates various stages of solvation and relaxation processes for the electrons during thermalization. As the electron loses energy, it becomes slow enough at even 10 eV to start polarizing the phonon modes of water, which deepens and forms the trap [8]. This process produces quasi-free electrons (e^−^_qf_), pre-hydrated electrons (e^−^_pre_) and finally solvated electrons (e^−^_aq_). (ii) Quasi-free electrons—electrons in the conduction band, are termed as e^−^_qf_ having energy near 0 eV and were very recently found to rupture sugar-base bonds in ribothymidine (a model of DNA) [6]. (iii) Pre-hydrated electrons are best defined as electrons in the process of solvation that quickly undergo full solvation leading to the formation of e^−^_aq_. (iv) Solvated electrons: Within 10^−12^ s, e^−^_qf_ completely solvates and becomes e^−^_aq_ with a chemical potential (∆G) of –1.6 eV; see Table 1 and Scheme 1. It is well known from theory [19,20] and experimentation [21,22] that e^−^_aq_ does not cause strand breaking/bond dissociation in DNA but binds efficiently with nucleobases. The nucleobase anion radicals protonate and eventually result in molecular products such as dihydrothymine and dihydrocytosine [16]. These products contribute to clustered damage (Section 4.4), so are important for radiation-induced cell death.

Excitation of e^−^_aq_ produces the excited state (e^−^_p_)* via s → p* transition with transition energy 1.73 eV (see Table 1). In the spectroscopy of the solvated electron the pre-hydrated electron is often incorrectly associated with this excited state (e^−^_p_)*. In this vertical excited state the electron resides in the potential formed by the ground state arrangement of waters around the fully solvated electron. It requires fast relaxation of solvent around (e^−^_p_)* to produce e^−^_pre_ below the conduction band. We note that e^−^_pre_ only lives for a very short time, <500 fs, and becomes the solvated electron [10]. Recently, using femtosecond time-resolved laser spectroscopy, e^−^_pre_ has been shown not to cause DNA strand breaks [6,23,24].

In that context, the conduction band (CB) energy width is important to any estimate of the energy of e^−^_qf_ and e^−^_pre_ as these are produced in or near the CB. In fact, *V*_o_ value (the adiabatic energy of the conduction band’s lower edge relative to the vacuum level) has uncertainties [25,26] and has been reported in the following energy range in water: −0.5 < *V*_o_ < −1.5 eV [27,28,29,30]. For extrapolations of cluster ion data, Coe et al. reassessed the electronic properties of bulk water, such as band gap, conduction band edge, and vacuum level, and they determined *V*_o_ in water as −0.12 eV < *V*_o_ < 0.0 eV [25]. Using first principles calculations, Gaiduk et al. [31] estimated the electron affinity of liquid water and of its surface as between −0.1 and −0.3 eV. We consider these later values most appropriate for *V*_o._ The various *V*_o_ values reported actually describe an electron in different degrees of trapping before full adiabatic solvation which is at ca. −1.6 eV [25,26,32]. In the CB the electron wave function is more delocalized compared to that in deeper traps, and thus these CB electrons (e^−^_qf_) will have a higher reaction rate with DNA [30]. The transfer of quasi-free electrons to DNA subunits then can form transient negative ions of DNA which may cause bond cleavage via dissociative electron attachment [6]. A comprehensive diagram showing the various states of the electron during solvation (e^−^_qf_, e^−^_pre_, and e^−^_aq_) and excitation (e^−^_p_*, vertical detachment energy (VDE)) is presented in Scheme 1.

### 2.2. Structure of the Solvated Electron (e^−^_aq_)

The solvated electron is a key species in radiation chemistry, electron transfer processes, redox phenomena, electrochemistry and photochemistry [1,4,5,36,37,38]. Since its discovery over 50 years ago [34,39], the genuine structure of e^−^_aq_ is still a matter of active debate [1,32,36,37,40,41,42,43,44,45,46,47,48,49,50]. Due to the short life time and low concentration of e^−^_aq_, its structure is accessible only by transient measurements [51]. Applying moment theory and absorption spectra of e^−^_aq_, Bartels and coworkers [35,52] estimated the radius of gyration (R_g_) of an e^−^_aq_, characterizing the average size of the e^−^_aq_ wave function. Many other observables of e^−^_aq_ derived from experiments are UV [34,39] and IR spectra [53]; ESR, g-factor [54,55], and hyperfine couplings (HFCCs) [56,57]; and thermochemistry [58,59], redox potential [60,61], and vertical detachment energy (VDE) [62,63,64]. Due to a lack of direct structure determination using conventional experiments, quantum chemical methods were extensively applied to model the structure of e^−^_aq_ with a challenge to reproduce the observed properties [34,35,52,53,54,55,56,57,58,59,60,61,62,63,64]. In this context the main question, “Does the solvated electron reside in a cavity or is it delocalized over a region of enhanced water density?” is yet a topic of ongoing debate [1,12,32,41,42,43,44,45,46,47,48,49].

### 2.3. Cavity Model of e^−^_aq_

Some structural information about e^−^_aq_ was obtained from ESR spectroscopic experiments [40,56]. From the ESR spectral line shape of e^−^_aq_, it was suggested that the electron is trapped in a potential well (cavity) surrounded by symmetrically oriented water molecules [40]. Based on the ESR spectral data in an alkaline, aqueous glass at 77 K, Kevan [56] proposed a six-water-molecule-coordinated structure approximately arranged in an octahedral fashion around e^−^_aq_ each with one of its O–H bonds oriented towards the electron. This preliminary structural assumption (cavity model) has been extensively explored using quantum chemical calculations and molecular dynamics (MD) simulations [32,36,45,46,65,66,67,68,69].

More recently using a quantum mechanics/molecular mechanics (QM/MM) approach, Jungwirth and coworkers [46] simulated the structure and dynamics of the electron in bulk water. In the simulation, 32 water molecules, residing closest to the center of the simulation box, were treated quantum mechanically using density functional theory while the remaining 992 water molecules were treated by MM and the simulation was run for 10 ps. Their simulation showed that the excess electrons in the quantum subsystem localized to a cavity-like structure within 3 ps. From the analyses of the radial distribution functions (RDFs), they found that the cavity enclosing the e^−^_aq_ is enclosed by four water molecules. Also, the calculated VDE and R_g_ of the solvated electron in bulk water matched very well with the experiment (VDE = 3.3–3.6 eV [62,63,64] and R_g_ = 2.48 Å [35]).

Several cavity-forming MD simulations based on electron-water pseudopotentials [50,68] and a polarization model [48] reproduced the experimental VDE, UV-vis spectra, and R_g_ of the electron in the bulk solvent. Although those simulations satisfactorily predicted those properties and enriched our understanding about the cavity formation enclosed by approximately four water molecules, they did not predict other important properties (thermochemistry, ESR parameters, such as hyperfine couplings and g-values, and redox potential). Thus, some uncertainty about the optimal water structure around the solvated electron remained.

**Figure 1:**
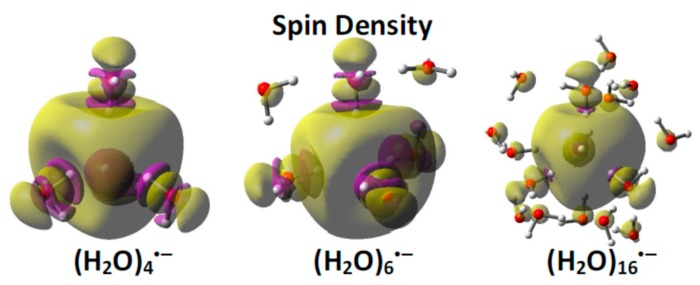
B3LYP-PCM/6-31G(2df, p) calculated spin density distributions of the excess electron in (H_2_O)_4_, (H_2_O)_6_ and (H_2_O)_16_. The yellow surface encloses 80% of the total spin density. Note that a significant amount of spin density is actually concentrated on oxygen atoms enclosing the cavity rather than between the water molecules. Reprinted with permission from reference [32]. Copyright, 2015, American Chemical Society.

Recently, using ab initio and DFT (density functional theory) methods and incorporating the effect of full solvation via the polarized continuum model, we have investigated the cavity model of the solvated electron [32]. In our approach, we considered four and six-water clusters ((H_2_O)_4_ and (H_2_O)_6_) to bind the excess electron. The initial geometry of (H_2_O)_4_ was arranged in a tetrahedral fashion while the initial geometry of (H_2_O)_6_ was arranged in an octahedral fashion, to bind the excess electron at the center. In those structures, one of the O−H bonds of each water molecule points toward the center of the cavity, as proposed by Kevan [40,56]. Full geometry optimization without any symmetry restriction by the B3LYP method resulted in convergence of these two structures to a tetrahedral conformation in which four water molecules formed the cavity and one O−H bond of each water pointed towards the center of the cavity. Frequency analyses confirmed that both the four and six water structures were at energy minima, as all the frequencies were positive. Those geometries were further optimized using different quantum chemical methods and large basis sets to test the stability of the tetrahedral structure. We also found that inclusion of additional water layers on the tetrahedral structure does not substantially change the result. The calculated spin density distributions of (H_2_O)_4_^•−^, (H_2_O)_6_^•−^, and (H_2_O)_16_^•−^ are shown in Figure 1. From a spin density distribution, we see that ca. 80% of the total spin is localized in the cavity while a significant spin density is localized on the oxygen atoms of each of the four core water molecules enclosing the cavity. The spin density distribution of e^−^_aq_ in bulk solvent derived from an MD simulation, carried out by Jungwirth and coworkers [46], showed that ca. 41% spin density resides in the cavity, while 24% resides on the first solvent shell, and 35% resides beyond the first solvent shell. Since spin density distribution is a measure of the extent of e^−^_aq_ localization, we note that our model shows dominant localization of e^−^_aq_ (ca. 80%) in the cavity, while Jungwirth’s [46] model shows only moderate localization of e^−^_aq_ in the cavity (ca. 40%). We note the MD calculations of Jungwirth and coworkers are room temperature simulations in which the water dynamics allow the electron to access a variety of states. Our calculations focus on the lowest energy configuration but calculate thermodynamic values at 298 K.

Further, to test the validity of our small tetrahedral cavity model of e^−^_aq_ [32], we calculated the various properties of e^−^_aq_ and found that our model was indeed successful in predicting the experimental properties: (a) Radius of gyration derived from the optical spectrum. (b) Vertical detachment energy. (c) Resonance Raman and the frequency red shift of the water O−H stretch. (d) Hyperfine couplings for electrons trapped in low temperature aqueous glasses. (e) ESR g-factor. (f) The experimental solvation free energy was correctly calculated, and a positive solvation entropy was predicted.

**Figure 2:**
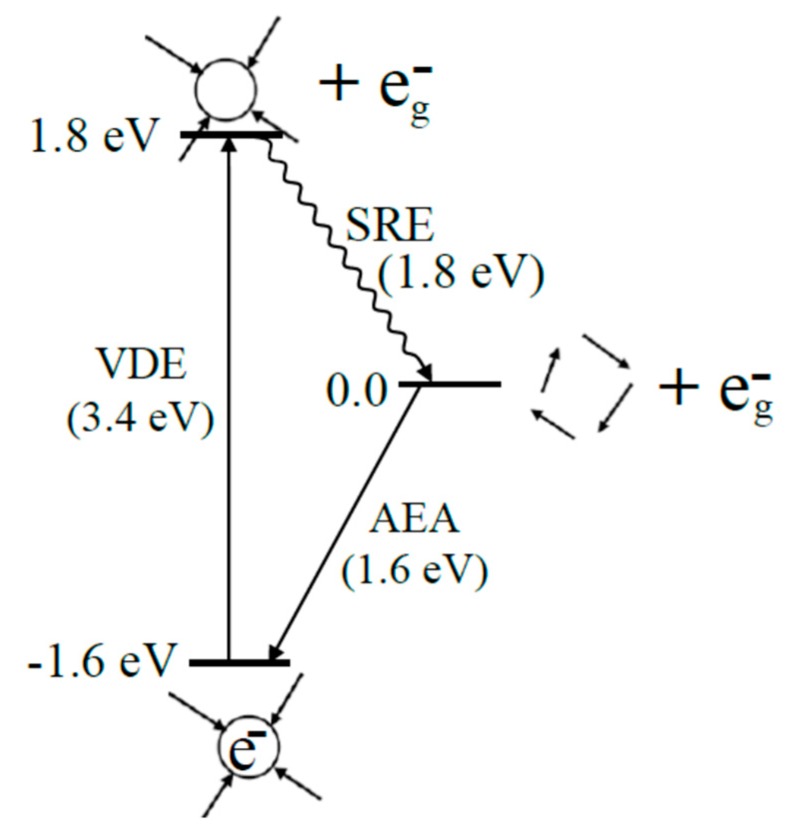
Schematic diagram showing the energy cycle for the adiabatic electron affinity (AEA), the photo-ejection of the solvated electron (e^−^_aq_) into the gas phase (VDE), and the solvent reorganization to equilibrated water (SRE = 1.8 eV). For the VDE, the water molecules have the same geometry for the upper state as in the ground state. The difference between AEA and VDE gives the value of SRE (solvent reorganization energy). The wavy line indicates the solvent reorganization. The SRE shows the solvent after photo-ejection of the electron is unstable by ca. 1.8 eV over a stable water phase geometry, see references [25,31]. Reprinted with permission from reference [72]. Copyright, 2016, American Chemical Society.

Similar to the 4-H_2_O tetrahedral cavity-model of e^−^_aq_ [32], Ambrosio et al. [70] proposed a distribution of the number of water molecules around the cavity with a slight predominance of a 5-H_2_O-coordinated e^−^_aq_ structure derived from their DFT-based MD simulation, including 64 and 128 water molecules. The simulation produced 34%, 44%, and 15% 4-H_2_O-, 5-H_2_O-, and 6-H_2_O-coordinated structures, respectively. Their conclusion was based on MD simulation trajectories only and those authors did not attempt to check with rigorous quantum chemical calculations that the 5-H_2_O structure is really more stable than the other conformations. In this context, between the two structures with six waters, (i) the middle structure shown in Figure 1, which has four waters around the electron and two in the next shell, and (ii) the octahedral 6-H_2_O cavity model of Kevan, the first (middle structure in Figure 1) was found to be more stable than the Kevan structure by ca. 10 kcal/mol, and the calculated vibrational frequencies were negative for the Kevan structure. The calculation was done using the B3LYP-PCM/6-31++G(2df,p) method [32]. Also, very recently, Walker and Bartels [71] calculated the structure of e^−^_aq_ in methanol considering 4–6 methanol molecules forming the cavity, using DFT and ab initio approaches. They considered three conformations for a 6-methanol cluster: (i) Tetrahedral, (ii) bipyramidal and (iii) octahedral, and they found that the tetrahedral structure with two methanols in the next solvent layer was more stable than the bipyramid and octahedron conformations. The properties calculated by Ambrosio et al. [70] of the 5-H_2_O-coordinated e^−^_aq_ are: (i) The absorption spectrum has peak at 1.75 eV. (ii) AEA (adiabatic electron affinity) = 1.28 eV, and (iii) VDE = −3.45 eV [70]. Their calculated AEA of 5-H_2_O-coordinated e^−^_aq_ (1.28 eV) is small in comparison to the experimental value (1.6 eV) [32,33]. Their comprehensive energy diagram [70] of the energy levels of excess electrons in liquid water is misleading as it places the VDE (−3.45 eV) lower than the AEA (1.28 eV) [25,31,38,72]; see Figure 2. Note that both VDE and AEA have the same ground state energy.

A very recent MD simulation of e^−^_aq_, based on correlated wave function theory (second-order Møller–Plesset perturbation theory (MP2)) was carried out by Wilhelm et al. [45], including 47 water molecules. The two trajectories were run for 2 ps and they found a delocalized nature for e^−^_aq_ at 0 ps, and a 5-coordinated cavity at 0.5 ps which converts to 4-coordinated cavity at 1 ps. From their simulation, they concluded that a four-coordinated tetrahedral cavity is the most stable configuration, since once formed it does not transform to cavities of different types, and they support the tetrahedral cavity model [32] made up of four water molecules as the likely dominant arrangement.

A schematic diagram showing the energy cycle for the adiabatic electron solvation and the photo ejection of the solvated electron (e^−^_aq_) into the gas phase (VDE) is shown in Figure 2. We note that the VDE and AEA of e^−^_aq_ are 3.4 [62,63,64] and 1.6 eV [32,72], respectively. These two quantities (AEA and VDE) of e^−^_aq_ are well-defined in the literature: (i) The AEA measures the binding energy of a formerly gaseous electron when it becomes solvated in liquid water, and (ii) the VDE is a measure of the instantaneous energy needed to remove an electron from its aqueous electron ground state in liquid water to the gas phase without nuclear reorganization (see Figure 2). The VDE of e^−^_aq_ is incorrectly compared to the AEA (1.6 eV) of e^−^_aq_ in a number of studies [63,68,70,73]. As Figure 2 shows the VDE starts with a fully solvated electron and ejects the electron from the cavity without nuclear reorganization (Franck–Condon principle). Thus, the water is left in a nonequilibrium state 1.8 eV above the zero of energy which is reached only after nuclear relaxation. Surprisingly the solvent reorganization energy is larger than the AEA.

### 2.4. Non-Cavity Model

Recently, Larsen, Glover and Schwartz [41] developed a new electron-water pseudopotential, including attractive and repulsive features of oxygen and hydrogen, and predicted a delocalized structure of e^−^_aq_ which occupies a ca. 10 Å-diameter region of enhanced water density. This non-cavity model is completely different from the well accepted cavity model of e^−^_aq_ and has been referred as the LGS model [1,44,47]. The LGS model does predict R_g_ = 2.6 Å, absorption spectra [41,42], and the absence of polarization anisotropy in excited states as observed in the transient hole-burning measurements on e^−^_aq_ [74], which the cavity-forming pseudopotential models fails to predict. Also, the experimentally observed red shift of absorption spectra of e^−^_aq_ with an increasing temperature at constant density was only captured by the LGS model in comparison to cavity-forming pseudopotential models [43]. Subsequently, the LGS model was found to be inadequate in reproducing certain physical observables, particularly, VDE and partial molar volume [1,42,69,75] (see Table 1) and became controversial [1,44,47,69,75].

A comparison of the performance of the cavity and non-cavity (LGS) models of e^−^_aq_ is presented in Table 1. In Table 1, we considered electron-water pseudopotential based LGS [1,44,47] (non-cavity) and Turi–Borgis (TB) [67,68] (cavity) models of e^−^_aq_ along with the tetrahedral water (4-H_2_O) [32] cavity model considered using DFT and ab initio quantum chemical approaches, including a polarized continuum model of solvation. From a detailed comparison of cavity versus non-cavity models of e^−^_aq_ in reproducing the experimental observables, it is evident from Table 1, that the LGS non-cavity model reproduces R_g_ and the absorption spectra’s maximum well. However, the cavity model of TB reasonably reproduces R_g_, the absorption spectrum maximum (blue shifted by 0.2 eV from experiment), and the linear temperature dependence of the spectral peak position (dE^MAX^/dT). In addition, the partial molar volume (V) is well reproduced (+31 ± 12 cm^3^/mol; exp = 26 ± 6 cm^3^/mol [75,76]) by the TB model. The partial molar volume is poorly predicted by the LGS model as it is highly negative (−116 ± 27 cm^3^/mol) [42]. In comparison to the LGS and TB models, the tetrahedral water (4-H_2_O) cavity model [32] is able to better reproduce a number of observables as clearly evident from Table 1.

## 3. Reaction of e^−^_aq_ with DNA Bases

### 3.1. Simulation Studies of ab Initio Molecular Dynamics (MD)

In this section, we focus on exploring the reaction of e^−^_aq_ with DNA/RNA bases carried out using an ab initio MD simulation and the prediction of accurate reduction potentials (*E^o^* versus absolute standard hydrogen electron potential, SHE) of the nucleobases calculated using high level quantum chemical Gaussian 4 (G4) theory.

**Figure 3:**
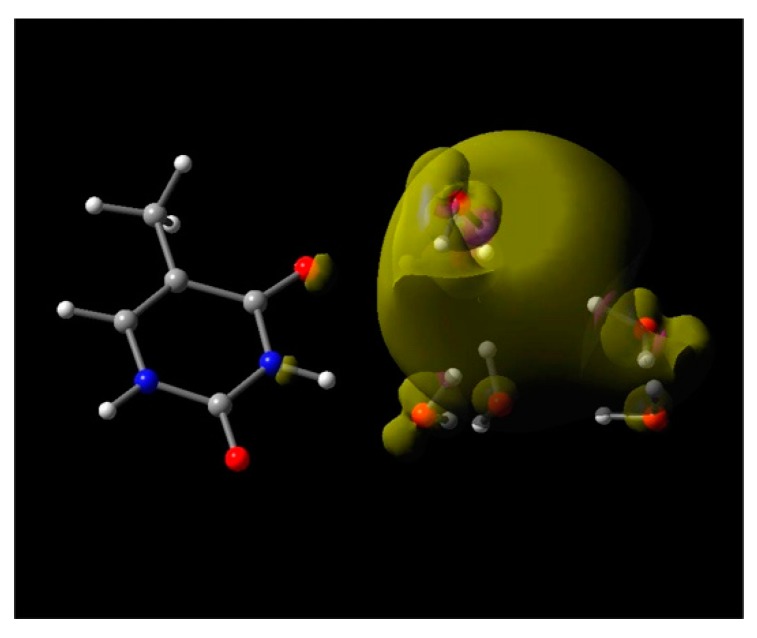
Total spin density plot of thymine (T)–6H_2_O^•−^. The structure is optimized to a local minimum using the B3LYP-PCM/6-31++G** method. Reprinted with permission from reference [72]. Copyright, 2016, American Chemical Society.

The reaction of e^−^_aq_ with the DNA/RNA bases has long been studied using pulse radiolysis experiments that reported near diffusion-controlled reaction rates (3.8 × 10^9^ to 1.8 × 10^10^ M^−1^·s^−1^; of e^−^_aq_ with nucleobases [5,13,77] (see Table 10.6 in reference [13]). Recently, this experimental fact has been disregarded by Abel and coworkers [37,63,78] and they proposed that e^−^_aq_ cannot reduce nucleobases. They argued that the electron affinities of solvated nucleobases are substantially lower (ca. 1.7–2.3 eV [79,80]) than the VDE (3.3 eV) of e^−^_aq_ which was considered as the binding energy by them [37,63,78]. That involved a misreading of the electron energy levels in water, as described earlier (Figure 2). Nevertheless, to verify by theory that e^−^_aq_ can reduce nucleobases (Adenine (A), guanine (G), cytosine (C), thymine (T), and uracil (U)), we modeled e^−^_aq_ in a cavity enclosed by 4H_2_O arranged in a tetrahedral conformation, as proposed by us [32], near each base (4H_2_O-base)^•−^ and fully optimized the structure by B3LYP/6-31++G**, including the PCM solvation model (B3LYP-PCM/6-31++G**) [72]. The spin density plot of the optimized (4H_2_O-base)^•−^ structure clearly showed that e^−^_aq_ is completely localized in the cavity enclosed by 4H_2_O in the tetrahedral arrangements and characterizes the structure as the 4H_2_O^•−^-base. Further, we added two more water molecules hydrogen bonded to the four-water cavity and completely optimized the structure (6H_2_O-base)^•−^ using B3LYP-PCM/6-31++G**. Again, the spin density plots characterize the structure as the 6H_2_O^•−^-base. The spin density plot of optimized thymine (T)–6H_2_O^•−^ is shown in Figure 3 as an example.

Figure 3’s static picture of a base interacting with e^−^_aq_ is in direct contrast with the experiment that all nucleobases react with diffusion-controlled rates with e^−^_aq_ [13]. The spin density plot, shown in Figure 3, seems anomalous, since the stable position for the electron is actually on the thymine, not in the water cavity as shown in Figure 3. However, this metastable state converts to the stable base anion radical formation (T–6H_2_O^•−^ → T^•−^-6H_2_O) when MD simulations induce the electron transfer as described below. In the MD simulation optimized base-6H_2_O^•−^ structures were used as the initial structures; see reference [72] for details.

**Figure 4:**
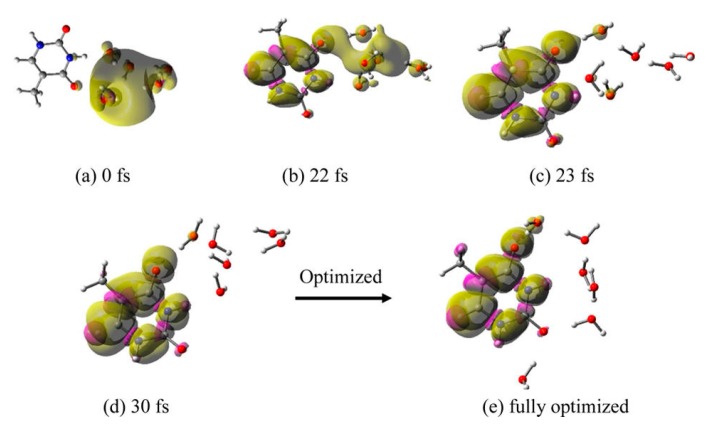
Total spin density plot of T–6H_2_O^•−^ at different times during MD simulation: (**a**) 0 fs (initial optimized starting structure for simulation), (**b**) 22 fs, (**c**) 23 fs, (**d**) 30 fs, and (**e**) fully optimized structure. Structure (**e**) is stabilized by -0.9 eV over structure (**a**). Reprinted with permission from reference [72]. Copyright, 2016, American Chemical Society.

(i) T–6H_2_O^•−^: The total spin density plot calculated for different structures present at various times during the MD simulation trajectory is shown in Figure 4. From the spin density plot of T–6H_2_O^•−^, we see that e^−^_aq_, which was initially (0 fs) localized in the cavity near thymine (base), starts transferring to thymine with time. At 22 fs, e^−^_aq_ is delocalized on both thymine and water and at 30 fs it is completely localized on the thymine. Considering the structure at 30 fs (Figure 4d), we completely optimized the structure by B3LYP-PCM/6-31++G**. In the optimized structure (Figure 4e), the waters are arranged near N1 to O4 sites in a hydrogen-bonded fashion. The optimized structure (Figure 4e) is more stable than the initial structure at 0 fs (Figure 4a) by 0.9 eV.

**Figure 5:**
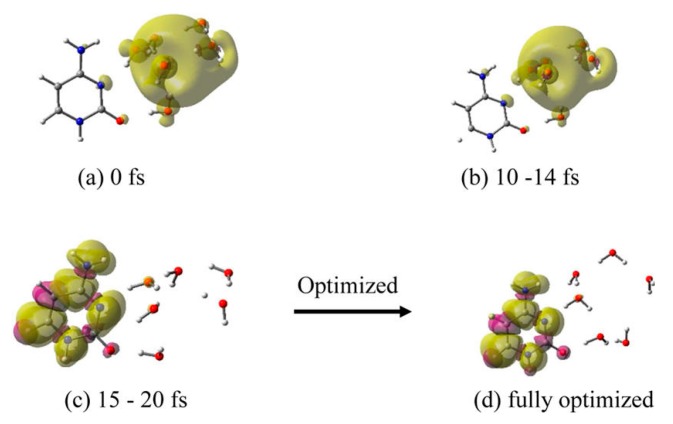
Total spin density plot of C-6H_2_O^•−^ at different times during MD simulation: (**a**) 0 fs (initial optimized starting structure for simulation), (**b**) 10–14 fs, (**c**) 15–20 fs, and (**d**) fully optimized structure. Structure (**d**) is stabilized by −0.64 eV over structure (**a**). Reprinted with permission from reference [72]. Copyright, 2016, American Chemical Society.

**Figure 6:**
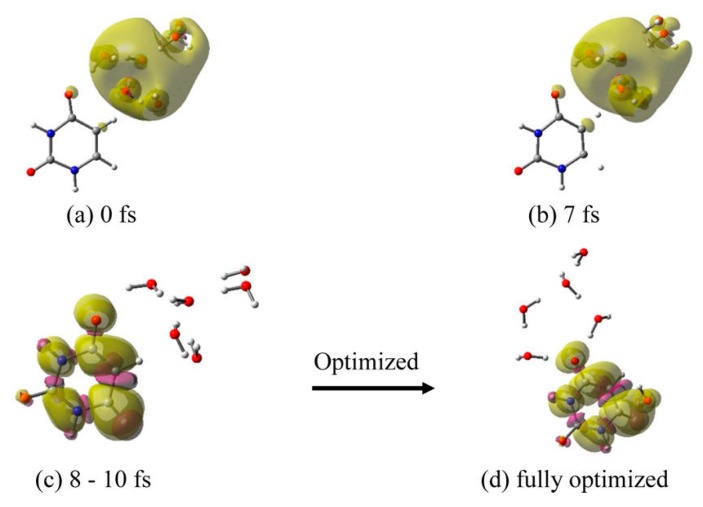
Total spin density plot of uracil (U)–6H_2_O^•−^ (6H_2_O^•−^ placed near O4-C5 site of uracil) at different times during MD simulation: (**a**) 0 fs (initial optimized starting structure for simulation), (**b**) 7 fs, (**c**) 8–10 fs, and (**d**) fully optimized structure. Structure (**d**) is more stable by 0.93 eV over structure (**a**). Reprinted with permission from reference [72]. Copyright, 2016, American Chemical Society.

**Figure 7:**
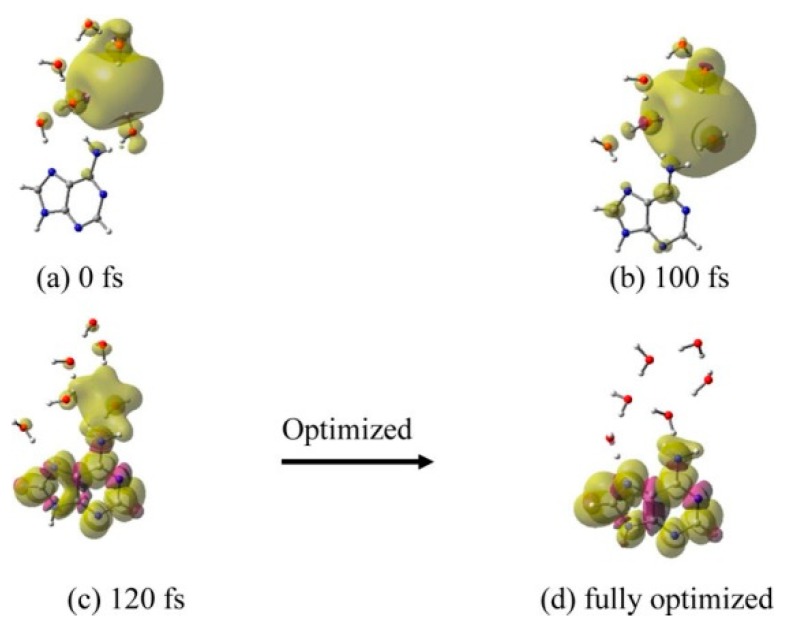
Total spin density plot of adenine (A)–6H_2_O^•−^ at different times during MD simulation. (**a**) 0 fs (initial optimized starting structure for simulation), (**b**) 100 fs, (**c**) 120 fs, and (**d**) fully optimized structure. Structure (**d**) is stabilized by −0.25 eV over structure (**a**). Reprinted with permission from reference [72]. Copyright, 2016, American Chemical Society.

The excess electron attachment to thymine solvated by 64 waters was studied by Smyth and Kohanoff using an ab initio CP2K MD simulation [81]. They also found that within 25 fs the excess electron is completely localized on thymine. The marked difference between our and their approaches was that we considered a completely localized e^−^_aq_ in a cavity interacting with thymine, while they considered the delocalized nature of an electron distributed on thymine and water molecules.

(ii) C–6H_2_O^•−^: An analysis of the MD simulation trajectory of C–6H_2_O^•−^, shows that up to 14 fs the electron resides in the cavity (Figure 5a,b) and that within 15–20 fs, it completely localizes on the cytosine base (Figure 5c). The structure at 20 fs (Figure 5c), which was fully optimized using the B3LYP-PCM/6-31++G** method, is shown in Figure 5d. This fully optimized structure was found to be more stable than the initial structure (Figure 5a) by 0.64 eV and water molecules arranged near the N4 to O2 atoms of cytosine.

(iii) U–6H_2_O^•−^: In uracil, we placed the 6H_2_O^•−^ (e^−^_aq_) at two sites (O4–N3 and O4–C5) and optimized both structures, and thereafter carried out MD simulations. For both the structures, the simulations showed that within 10 fs the solvated electron completely transfers to uracil. This is faster than thymine and cytosine; see Figure 4 and Figure 5. In Figure 6, we show the results of 6H_2_O^•−^ placed near the O4–C5 site. The fully optimized structure (Figure 6d) using the B3LYP-PCM/6-31++G** method, is found to be more stable than the initial structure (Figure 6a) by 0.93 eV. In the optimized structure (Figure 6d) five water molecules are localized around O4 in a hydrogen-bonding fashion while a sixth water molecule drifted towards the C6 atom of uracil and was stabilized via hydrogen-bond formation with the π_z_-MO on C6 of uracil. The initial structure in which 6H_2_O^•−^ was placed near O4–N3 site of uracil was found to be 4 kcal/mol less stable than the structure in which 6H_2_O^•−^ was placed near O4–C5 site of U; shown in Figure 6a

(iv) A–6H_2_O^•−^: From the MD simulation of adenine (A)–6H_2_O^•−^, we see that the electron does not transfer to adenine up to 100 fs except for only a very minute spin density builds up on adenine (Figure 7b). At 120 fs, e^−^_aq_ is delocalized on adenine (75% spin) and water molecules (25% spin); see Figure 7c. Full optimization of the structure at 120 fs results in structure in Figure 7d.

Optimization of the structure in Figure 7c converges to a structure with complete localization of the electron on adenine with one water molecule hydrogen-bonded to the N7 atom of adenine, while the other five waters are arranged near the NH2 of adenine. The structure from Figure 7d is stabilized by 0.25 eV over the structure from 7a [72]. Compared to C, T and U, the reaction of e^−^_aq_ with adenine is quite slow.

**Figure 8:**
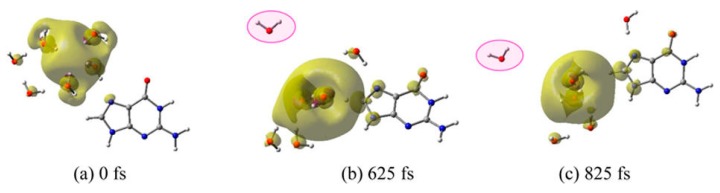
Total spin density plot of guanine (G)–6H_2_O^•−^ at different times during MD simulation. (**a**) 0 fs (initial optimized starting structure for simulation), (**b**) 625 fs, and (**c**) 825 fs. The 6H_2_O is located near the N7–C8 site of guanine. Reprinted with permission from reference [72]. Copyright, 2016, American Chemical Society.

(v) G–6H_2_O^•−^: For guanine, we considered three structures by placing 6H_2_O^•−^ (e^−^_aq_) near to the N7−C8, O6−N7, and O6−N2 sites of guanine, respectively. For all three structures stated above, MD simulations were carried out for 1.25 ps with a time step of 0.25 fs. From the analyses of the MD trajectories for each structure, it was found that e^−^_aq_ resides in a cavity and only a small fraction; ca. 20% of the total spin is transferred to guanine up to 1.25 ps. In Figure 8, we present the total spin density plots calculated at 0, 625 and 825 fs for the structure in which 6H_2_O^•−^ is located near to the N7−C8 site of guanine.

From simulation, it was found that, with time, a water molecule (highlighted in pink) moves away from guanine, and interacts weakly with the system in question; see Figure 8b,c. To take into account the full interaction of all water molecules with guanine, the structure at 625 fs was modified. The distant water molecule (pink highlighted) at 625 fs was manually moved towards O6 of guanine to stabilize the anion radical without affecting the configuration of the rest of the system present at 625 fs (Figure 8b).

**Figure 9:**
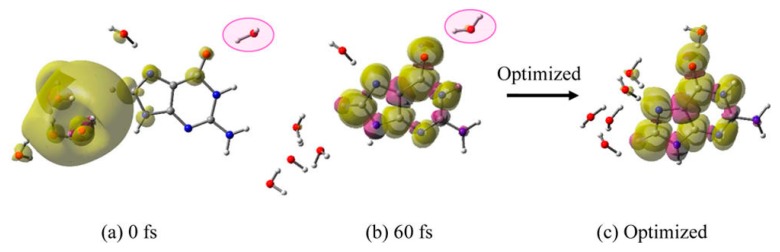
Total spin density plot of G–6H_2_O^•−^ at differing times during MD simulation: (**a**) 0 fs, (**b**) 60 fs and (**c**) Optimized structure. The 0 fs structure is generated from the 625 fs simulation (Figure 8b) by moving one water molecule (pink highlighted) to hydrogen bond with O6 of guanine. The structure in Figure 9c is 0.19 eV more stable than the structure shown in Figure 8a. Reprinted with permission from reference [72]. Copyright, 2016, American Chemical Society.

This modified structure was used for MD simulations and the results are presented in Figure 9. From Figure 9, it is evident that, with the new location of a single water, within 60 fs, e^−^_aq_ is completely transferred to guanine as the total spin density localizes on guanine. The optimized structure (Figure 9c) is more stable by 0.19 eV than the initial structure given in Figure 8a. This result emphasizes that for guanine the hydrogen bonding water network is necessary to stabilize the guanine radical anion for fast electron transfer from cavity to guanine.

Summary: From MD simulations of e^−^_aq_ interacting with DNA/RNA bases, it is evident that all the natural bases bind the e^−^_aq_ efficiently. These simulations support the well-established experimental fact that e^−^_aq_ reacts with all nucleobases at a diffusion-controlled rate [5,13,77]. For U, T and C the electron transfer from the cavity to base is complete on a 10–30 fs time scale. For A the transfer is complete within 120 fs. However, for G, a more extensive water hydrogen-bonding network around guanine is necessary to capture the e^−^_aq_. The more rapid capture by the pyrimidines is clearly a result of their higher electron affinities. The electron affinities of the purines are so close to that of the solvated electron that the small driving force takes additional time for solvated electron waters to reorganize, to allow for electron capture by the base. We note those time scales for the encounters of e^−^_aq_ with nucleobases, leading to its addition, are too short to affect the diffusion controlled rate constants of the reactions of e^−^_aq_ with nucleobases. The relative stability of the solvated base anion radicals is in the order G^•−^–6H_2_O < A^•−^–6H_2_O < C^•−^–6H_2_O < T^•−^–6H_2_O < U^•^ –6H_2_O which is in excellent agreement with the trends obtained from the experimental *E^o^* versus SHE [72]. In addition, the reduction potentials of nucleobases were also calculated to strengthen the conclusion drawn from the MD simulations.

### 3.2. Base Reduction Potentials

The reduction potentials of A, T, G, C, and U were experimentally determined by Seidel et al. [82] using pulse polarography and cyclic voltammetry in N, *N*-dimethylformamide (DMF; ε = 37.22). Using the Gaussian 4 (G4) level of theory, the one-electron reduction potentials (*E^o^*) of A, T, G, C, and U were calculated in three environments: (a) In DMF, (b) in aqueous phase, and (c) with clusters of two and three water molecules. The full effect of solvent (DMF and water) was taken into account via the polarized continuum model (PCM) solvation model. The standard one-electron reduction potential *E^o^* versus SHE is calculated as:

(1) $$E^{o}=\frac{-\Delta G_{Sol}^{o}}{F}-\mathrm{SHE}$$

Where SHE is the absolute standard hydrogen electrode potential and SHE = 4.44 V was used. However, the reported values of the SHE vary from 4.28 to 4.44 V; for details, see reference [51,72]. F is the Faraday constant equal to 23.061 kcal/(mol·volt) or 1 eV/volt. The ratio (−Δ*G^o^_sol_*/F) is in volts. *E^o^* of one-electron reduced DNA/RNA bases are calculated in the appropriate solvent using the direct method [83]; i.e., without considering the thermodynamic cycle. The overall reaction of the solute in the solvent with the gas phase electron (*e^−^_g_*) is:

(2) $$M_{sol}+e_{g}^{-}\overset{\Delta G_{sol}^{o}}{\to }M_{sol}^{•-}$$

And the free energy is calculated using Δ*G^o^_sol_* = *G^o^* (*M^•−^_sol_*) − *G^o^* (*M_sol_*) − *G^o^* (*e^−^_g_*). The free energy of the gas phase electron, *G^o^* (*e^−^_g_*), of −0.867 kcal/mol is obtained from Fermi–Dirac statistics [84]. In the calculation of *E^o^*, the standard states chosen are 1 atm, 1 mol, and 298 K, respectively.

The calculated *E^o^s* of A, T, G, C, and U in DMF and in water by the G4 method and by B3LYP-PCM/6-31++G** in water along with the experimental values in DMF are presented in Table 2. From Table 2, we see that the G4 calculated *E^o^* values of G, A, C, T, and U in DMF are in reasonable agreement with those estimated experimentally by Seidel et al. [82] in DMF, and we find a mean unsigned error (MUE) of 0.22 V. Using the M06-2X/6-31++G(d,p) method, Lewis et al. [85] calculated the *E^o^s* of G, A, C and T in water as −3.31, −2.86, −2.41, and −2.31 V; and the MUE of the experiment is 0.27 V. Crespo–Hernández et al. [86] used gas-phase vertical electron affinities of G, A, C, and T, calculated using the B3LYP/6-311+G(2df,p)//B3LYP/6-31+G* method to estimate the *E^o^* of G, A, C and T as ca. −3.0, −2.71, −2.56, and −2.32 V, respectively, in acetonitrile (ε = 36.64) with MUE with respect to the experiment of 0.2 V.

**Figure 10:**
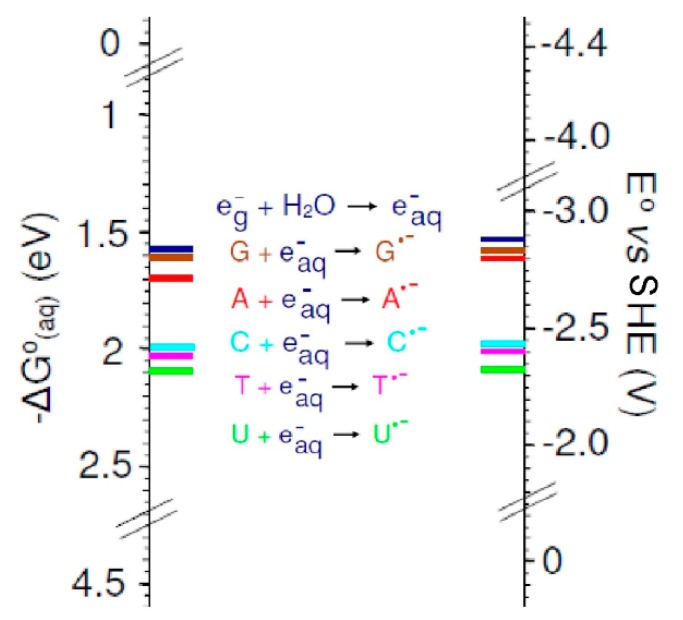
A comparison of the G4-calculated free energies of electron addition to water and various DNA bases, Δ*G*^o^_(aq)_ (eV), along with *E^o^* versus SHE of all the bases and solvated electron indicated by colored bars on scales. The scales differ by 4.44 V. Reprinted with permission from reference [72]. Copyright, 2016, American Chemical Society.

From Table 2, it is clear that G4 calculated *E^o^s* of nucleobases in DMF or in water lie in the order G < A < C < T < U, which is in agreement with experimentation, and with a reasonable fit quantitatively. The B3LYP-PCM/6-31++G** calculated that the *E^o^s* of the bases are in close agreement with those calculated by G4 method. The small discrepancy between experiment and these G4 and B3LYP calculations is due to the lack of solute–solvent hydrogen-bonding which is not considered in solvation models, such as PCM. In view of this, we placed two to three explicit water molecules around the bases in question and included PCM. We used the G4 method to calculate the *E^o^s* of A, T, G, C, and U and the results are given in Table 2. It is evident that the inclusion of explicit water molecules of hydration increase the reduction potentials (and electron affinities) of the various DNA bases and reduces the error in comparison to experiment significantly (MUE = 0.07 V). The G4 calculated of *E^o^* of guanine (−2.62 V, see Table 2) and the experimental estimate of <−2.76 V are near to the *E^o^* of e^−^_aq_ (−2.87 V, see Table 1). Uracil is the most easily reduced among all the bases. A comprehensive diagram, based on the solvation free energy and one-electron reduction potential (*E^o^* versus SHE) of bases including e^−^_aq_ is shown in Figure 10.

## 4. Electron Reactions in Ion-Beam Irradiated DNA

### 4.1. Introduction

As with all ionizing radiation, energy deposition in matter from a high energy photon or ion-beam results in a cascade of secondary electrons and excitations which are distributed spatio-temporally in a nonhomogeneous track [87,88,89,90,91]. Various treatments of the stochastic processes involved in radiation deposition and subsequent processes have been formulated and have been reviewed extensively [87,88,89,90,91]. In the above processes electrons in various energy states (LEE, solvated electrons, etc.) are produced in copious amounts and account for much of the damage done by the radiation [8,18].

**Scheme 2:**
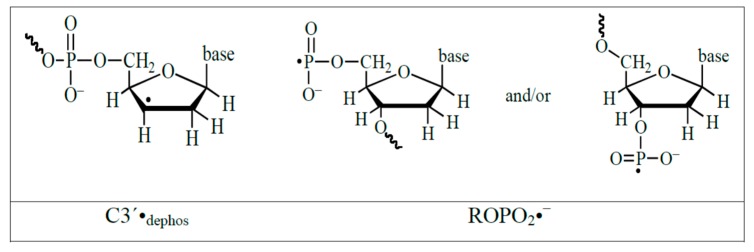
Structural formulae of the radicals formed in DNA via dissociative electron attachment.

**Scheme 3:**
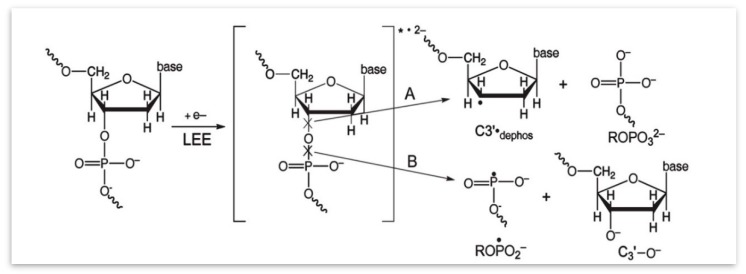
Formation of ROPO_2_•− and C3′•_dephos_ by low energy electrons. Attachment of a low energy electron (LEE) results in the formation of a transient negative ion (TNI), in which bond cleavage of the C3′–O or the P–O bond occurs. If the reaction occurs through channel A, the C3′•_dephos_ radical and a diamagnetic species are formed. If the reaction uses channel B, ROPO_2_•− and a different diamagnetic species form. In both cases, a bond in the sugar-phosphate backbone is broken, resulting in an immediate strand break. Approximately 95% of the TNI decomposes through channel A and 5% through channel B. (Used with permission by Radiation Research, at the 2019 Radiation Research Society, from reference [96]).

For many years, the electron was thought to be of secondary importance in the radiation chemistry of DNA [13,92]. Indeed it is now well-established from the reaction of e^−^_aq_ with various DNA bases (Section 3) that e^−^_aq_ capture by the electron-affinic bases—cytosine and thymine—occurs rapidly in DNA, and the resultant thymine and cytosine anion radical (T^•−^ and C^•−^) upon protonation form the C6-hydrogen adduct radicals which lead to products (e.g., dihydrothymine and dihydrocytosine) that usually are easily repaired via base excision repair pathways [13]. Thus, oxidative processes (i.e., the pathways that involve base and backbone cation radicals) were considered to be the primary damage pathway in radiation damage to DNA [93]. However, 21st century discoveries of the ability of LEE to rupture bonds has brought a revived interest in the critical role that electrons (LEE, e^−^_pre_, e^−^_aq_ (Section 2) have to play in the radiation damage of DNA [6,8,18,30,93,94,95,96]. In addition, with the recognition that clustered damage which includes a double strand break (DSB) may be especially lethal to a cell [13], damage initiated by electron attachment to a DNA base as well as to the backbone plays a role in the damaging effects of radiation [6,7,17,80,93,94,95,96]. The LEE mechanisms for formation of radicals shown in the Scheme 2 and Scheme 3 will be discussed in this section.

### 4.2. Low Energy Electron-Mediated Radicals in Ion-Beam Irradiated DNA

**Figure 11:**
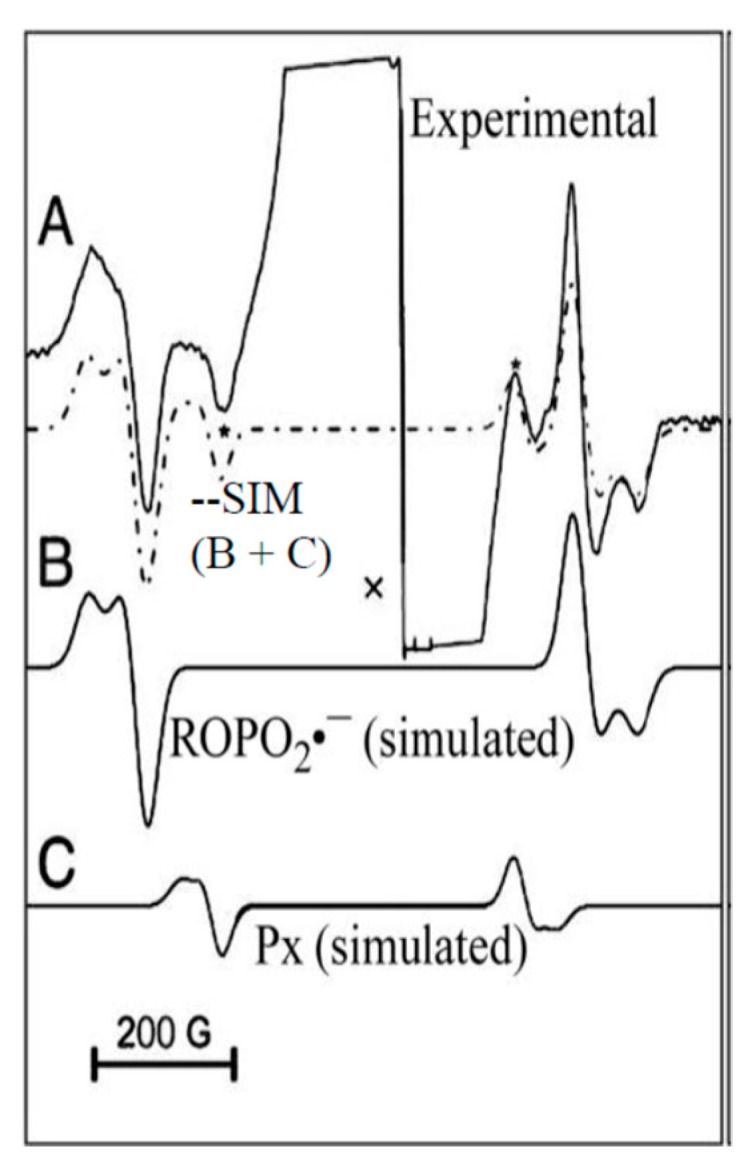
(**A**) Experimental spectra at 77 K of phosphorus-centered radicals in Kr-86 ion beam irradiated (at 77 K), hydrated DNA. The dashed line is the sum of the simulated spectra in B and C. (**B**) Simulated spectrum (for parameters see text) of the main phosphorous-centered radical, ROPO_2_•−. (**C**) Simulated spectrum of unknown phosphorous-centered radical (P_x_). (Adapted from reference [96] with permission from Radiation Research, at the 2019 Radiation Research Society).

(i) ROPO_2_•−: Samples of hydrated DNA which are subjected to ion-beam irradiation display a weak but recognizable electron spin resonance (ESR) spectrum that arises from the presence of a phosphorus-centered radical (Figure 11). The solid line in Figure 11A is the ESR spectrum of Kr-86 ion-beam irradiated hydrated (12 D_2_O/nucleotide) salmon sperm DNA [96] irradiated to a dose of 47.9 Mrad at a LET (linear energy transfer) of 2183 ± 50 keV/µm. In Figure 11B, the simulated spectrum of ROPO_2_•− (Scheme 2) using parameters A_‖_ = 793 G, A_⊥_ = 631 G, g_‖_ = ca. 2.0028, g_⊥_ = ca. 2.0060, and a Lorentzian linewidth of 35 G is shown. Figure 11C shows the spectrum of an as yet unknown underlying phosphorus-centered radical (P_x_) with two line components visible in the experimental spectrum (stars). The simulated spectrum for P_x_ (Figure 11C) is presented at 20% of the intensity of that of ROPO_2_•− (see reference [96] for details). The dashed line in Figure 11A is the sum of the simulated spectra in B and C, and very closely matches the experimental spectrum, providing good evidence of the presence of ROPO_2_•− in Kr-86 ion-beam irradiated DNA. In Figure 11, the centrally located “X” is at *g* = 2.00; the centrally located, large, off-scale component arises from the base radical and sugar radical spectra shown elsewhere in this work.

**Figure 12:**
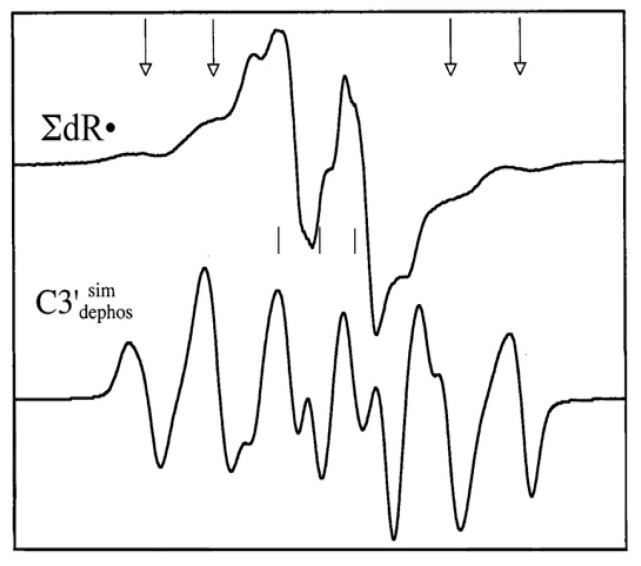
Experimental ESR spectrum of sugar radicals in Kr-86 ion beam irradiated DNA (∑dR•) and simulated spectrum of C3′•_dephos_ using parameters given in narrative. The arrows indicate the positions of the outer four lines of C3′•_dephos_ in the experimental spectrum (top). Adapted from reference [98] with permission from Radiation Research, 2019 Radiation Research Society.

Spectra for ROPO_2_•− are also visible in O-16 and Ar-36 ion-beam irradiated DNA [97,98]. After the discovery of ROPO_2_•− in ion-beam irradiated samples, a diligent search revealed its presence in γ-irradiated samples, but at about 1% of the concentration of that in an ion-beam, sample similarly irradiated [99]. Because of its low concentration in γ-irradiated samples, the formation of this radical is clearly an ion-beam core phenomenon, likely due to the high deposition of energy and large number of electrons in the ion-beam core, which, together facilitate formation of an excited state of the transient negative ion, discussed in Section 4.3.

(ii) C3′•_dephos_: The central portion of the ESR spectrum of Ar-36 ion beam irradiated DNA reveals a second radical formed from LEE, in concert with ROPO_2_•−, the C3’•_dephos_. Figure 12 shows the spectrum of the neutral sugar radicals found in Ar-36 irradiated DNA, ∑dR•, derived by subtraction of a low dose sample from a high dose one (see reference [98] for details). A simulated spectrum of the radical which gives rise to the outer line components is shown as well. This radical has four proton couplings and its multiline spectrum extends over ca. 100 G. The proton hyperfine couplings used in the simulation were: (***A_xx_***, ***A_yy_***, ***A_zz_***) = (−11, −23.4, −34 G) for one anisotropic alpha proton, and, for three beta protons with isotropic couplings, 26.9 G (1H), 34.9 G (1H), and 49.1 G (1H). In addition, anisotropic g-values, *g_xx_* = 2.0036, *g_yy_* = 2.0023, and *g_zz_* = 2.0044 and an isotropic linewidth (5.6 G) were used for this simulation. We note here that the mechanism of formation of C3′•_dephos_ (Scheme 3) in LEE-mediated DNA damage has been supported from product analyses studies of LEE-induced damaged oligomers [100,101]. These studies show that the C3′–O and C5′–O bond cleavages are the predominant pathways, and P–O bond cleavage accounts for minor pathways for LEE-mediated DNA damage [100,101]. These results support the above-mentioned ESR results from ion-beam irradiated DNA. In addition, line components of C3′•_dephos_ have been observed via photoexcitation of 2′,3′-dideoxypurine and pyrimidine cation radicals (e.g., 5-methyl-2′,3′-dideoxycytidine cation radical (5-Me-2′,3′-ddC•^+^), 2′,3′-dideoxyadenosine cation radical (2′,3′-ddAdo•^+^) and one-electron oxidized 2′,3′-dideoxyathymidine (2′,3′-dd T(-H)•)) [102].

### 4.3. Mechanism of Formation of ROPO_2_•− and C3′•_dephos_ via LEE-Mediated Dissociative Electron Attachment

The formation of both ROPO_2_•− and C3′•_dephos_ is attributed to LEE through electron attachment to the DNA, followed by bond cleavage at the C3′–O bond (Scheme 3).

**Figure 13:**
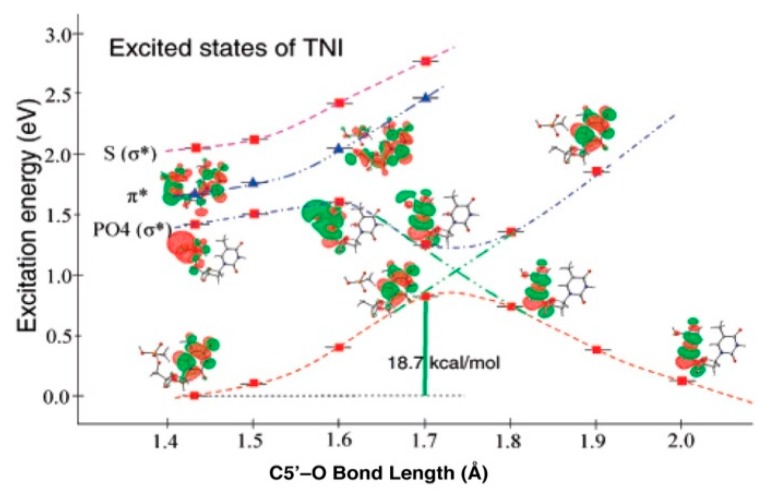
The lower curve shows the energy of the 5′-dTMPH^−^* TNI as the C5′-O bond is lengthened. Extension of the bond allows the TNI to move from the bound π ground state in which the SOMO is located largely on the thymine base to a dissociative σ(PO_4_)* state in which the SOMO on the phosphate group. Reprinted with permission from reference [103], copyright 2008 American Chemical Society.

The formation of strand breaks by LEE was first observed by the Sanche group [8,18,89,90,95] and the mechanism shown in Scheme 3 is consistent with these results. The site of LEE attachment and details of the reaction have been a source of interest. In order to investigate the rupture of a bond between a sugar carbon and oxygen, a time dependent DFT study of gas phase 5′-thymidine monophosphate (5′-dTMPH) shows that the LEE can add to the LUMO (lowest unoccupied molecular orbital) of the ground state of 5′-dTMPH, to give a π state in which the SOMO (singly occupied MO) formed localizes over the thymidine ring [7,103]. In the ground state of the TNI as the C5′–O bond is stretched, the energy increases and it is able to cross to a dissociative (PO_4_)* state. The bond cleavage reaction occurs with an 18.7 kcal/mol activation barrier (Figure 13) which effectively eliminates the ground state anion as a source of cleavage [7,103]. The likely, route shown in Figure 13, involves LEE addition to a vertical σ*-excited state localized on PO_4_ with transition energy (<1.5 eV) (π → σ(PO_4_)*). This results in a direct strand break. That calculation is consistent with the proposition that an excited state TNI is the intermediate species that is formed during the C5′–O bond cleavage. Given the high energy deposition in an ion-beam core, it is energetically possible for a LEE to directly attach to the phosphate group in DNA, which is already in a vibrationally excited state (Feshbach resonance), and undergo C5′–O bond cleavage [7,103]. We note here that these theoretically predicted results are supported by studies on the reactions of LEEs with 5′-dCMP in the gas phase [104]. These experiments established that LEE can induce single strand breaks by two routes: (1) Predominantly (60%) by direct localization on the phosphate moiety, and (2) electron localization on the C base and transfer of the excess electron to the phosphate backbone [104].

A further calculation on the full solvation effect of an aqueous environment on this reaction found that the dissociative σ*(PO_4_) state is blue-shifted towards higher energy and, thereby, LEE below 4 eV might not be able to initiate a strand break [19]. The authors state, “This clearly suggest that solvation of DNA anion would reduce the direct cleavage of DNA by LEEs in the energy range 0 to 4 eV” [19]. However, the vertical attachment and subsequent dissociation of the TNI takes place on the same time frame as solvation. For this reason, solvation effects will likely be less dominant than predicted in these study [19].

### 4.4. The Relevance of the Solvated Electron (e^−^_aq_)

Although it has long been recognized that e^−^_aq_ does not cause strand breaks in DNA in an aqueous environment [13,21,22,92], because of the effects of clustered damage, e^−^_aq_ are still relevant to the overall effect of ionizing radiation in DNA. Isolated base damage, single strand breaks, and even double strand breaks are not necessarily lethal to a cell, since most isolated damage is repairable through regular damage repair pathways. However, clustered lesions, in which strand breaks and/or base damage (see page 2) of some sort (including abasic sites) are in proximity, may indeed be lethal, since such complex damage sites may not be recognized by repair enzymes [13,105]. Ion-beam irradiation of hydrated DNA at 77 K results in the formation of the base radicals C(N3)H• (the reversibly protonated form of C^•−^) and T^•−^ in the penumbra of the track, through electron attachment to the bases [93,96], and these radicals may contribute to a clustered damage site. For instance, in Kr-86 ion-beam irradiated DNA, the pyrimidine anionic radicals constitute 42 ± 3% of the trapped base radicals at 77 K, with the remainder originating from the oxidative path [96]. Both C^•−^ and T^•−^ are formed by direct electron attachment to the bases due to the relatively high electron affinities of cytosine and thymine [13,106]. It should be noted that it was shown experimentally that not only aqueous electrons, but that e^−^_pre_ also adds to the pyrimidine bases to form 5,6-dihydropyrimidine products from the pyrimidine anion radicals [16]. In agreement, recent picosecond pulse radiolysis study confirmed that both aqueous electrons and prehydrated electrons can contribute to the formation of pyrimidine anion radicals but not to strand breaks [6,24]. The T^•−^ and C^•−^ form dihydrothymine and dihydrouracil, respectively, upon warming [16]. These base damage sites, in turn, may be contributors to a clustered damage site.

## 5. C5-Modified Pyrimidine Nucleosides as Radiosensitizers (Radiation Damage Enhancement Agents)

### 5.1. C5-Modified Pyrimidines as Radiosensitizers, a Short Summary

As the pyrimidines are more electron-affinic than purines [13,92,106], modified pyrimidine derivatives, for example, the C5-halopyrimidines are well-investigated as electron-affinic radiosensitizers in cancer radiotherapy. For example, cells incorporate 5-bromo-2′-deoxyuridine (5-BrdU) in their DNA almost as readily as thymidine and depending on the extent of 5-BrdU incorporation in cells, 5-BrdU has been shown to provide up to a 3–4 fold increase in radiation-induced cell killing [13,92,107,108,109,110]. Thus, it has long been recognized as a radiosensitizing agent with potential clinical applications [13,92,107,108,109,110]. However, owing to the toxicity of 5-BrdU, it did not show any increase in patient survival during phase III clinical trials and the trials were called off [107]. Thus, development of non-toxic and efficient pyrimidine C5-modified analog as radiosensitizers is of great interest [108,109,110]. The C5-substituted pyrimidine, 5-(phenylselenyl)methyl-2′-deoxyuridine was shown to induce oxygen-independent benzyl-type (5-CH_2_•) radical-mediated DNA inter-strand cross-linking [111] and apoptosis in cancer cells [112]. In addition, reactions of 5-thiocyanato-2′-deoxyuridine (dU-5-SCN) with radiation-produced prehydrated electrons lead to predominant cleavage of the S–CN bond to form the neutral uracil-C5-thiyl radical (dU-5-S•) [113] and the subsequent dU-5S-5S-dU dimer. Thus, dU-5-SCN could be a potential radiosensitizer by forming inter and intra-strand crosslinks and DNA-protein crosslinks through S-S dimer formation [113]. In fact, the corresponding 5-selenocyanato-2′-deoxyuridine (dU-5-SeCN) showed ca. 1.5 times more DEA-mediated degradation than 5-BrdU via formation of uracil C5-selenyl radical (dU-5-Se•) [114] and the subsequent dU-5Se-5Se-dU dimer. Both dU-5-SeCN and 5-tri-fluoromethanesulfonyl-2′-deoxyuridine (OTfdU) did not exhibit observable cytotoxicity in MCF-7 breast cancer cells in the absence of radiation and hence have shown promise to be employed as radiosensitizers [114]. In addition, several radiation chemical and biological studies have been carried out which show that radiation-produced electrons (LEE, e^−^_aq_) play an important role in the formation of DNA damage from electron-mediated radiosensitization employing other radiosensitizers, such as, cisplatin [115,116].

### 5.2. Formation of the Oxidizing Aminyl Radical under a Reductive Environment

Full characterization of the aminyl radical, RNH•, is extremely important for understanding the chemistry of biological molecules, since amine groups are common to DNA bases, amino acids, and other biomolecules [13,92,93]. Irradiation followed by ESR studies in homogeneous glassy solutions of DNA-bases, nucleosides, and nucleotides, as well as X-ray irradiation of single crystals of DNA-models at low temperatures, have established that DNA-heteroatom centered radicals, such as RNH•, are produced via deprotonation of purine or pyrimidine-base cation radicals (or holes) [3,13,17,92,93,117,118,119]. These observations are well-supported by pulse radiolysis, as well as flash photolysis studies of the cation radicals in aqueous solutions at ambient temperature [13,92,120]. Studies in our laboratory have established that RNH• plays an important role in the prototropic equilibria of DNA-base π-cation radicals (such as, guanine cation radical, cytosine cation radical, adenine cation radical) [3,17,93,117,121,122,123,124,125,126]. Apart from RNH• formation via deprotonation of base cation radicals, pulse radiolysis and product analyses studies have established that hydroxyl radical (•OH) can also produce RNH• in purines, via •OH addition to the C4=C5 double bond of the purine ring, along with subsequent water elimination, or via H-atom abstraction from the exocyclic amine group [13,92].

**Scheme 4:**
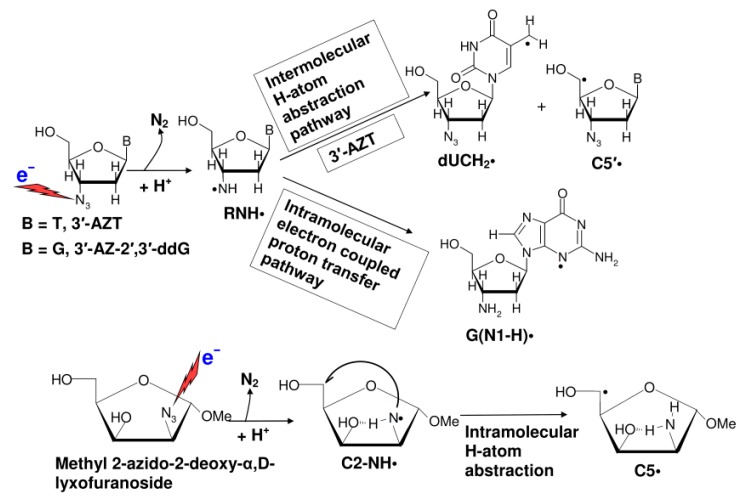
RNH• Formation and its reactions.

Employing ESR spectroscopy, we observed that in azido-substituted nucleosides, e.g., in 3′-azidothymidine (3′-AZT), radiation-mediated electrons lead to neutral aminyl radical (RNH•) production at the C3′-site of the sugar moiety at 77 K. The mechanism proposed is that electron addition to 3′-AZT results in the highly unstable azide anion radical (RN_3_•¯) which undergoes prompt N_2_ loss to form the nitrene anion radical (RN•¯). The nitrene anion undergoes rapid protonation of RN•¯ which results in a site-specific formation of a localized RNH• which is observed by ESR (Scheme 4) [126]. In 3′-AZT, RNH• subsequently undergoes a bimolecular H-atom abstraction both from the methyl group at C5 in the thymine base to give the allylic dUCH_2_• and from the C5′-atom to give C5′• of a proximate 3′-AZT (Scheme 4) [126]. For RNH• formed in azido-substituted pentofuranoses, it is found that sugar ring configuration influences the pathway and site of the H-atom abstraction reactions [127]. Sugar radicals, e.g., C5′•, are well-established as precursors of DNA-strand breaks [13,127]. Moreover, RNH• and base radicals (e.g., dUCH_2_•) can cause crosslink formation [13]. These lesions, eventually, lead to the induction of apoptosis of cancer cells [13,111,112,113,114]. Furthermore, ESR studies of 3′-azido-2′,3′-dideoxyguanosine (3′-AZ-2′,3′-ddG) present evidence of an intramolecular electron transfer process from the guanine base to the radiation-produced 3′-aminyl radical leading to the formation of one-electron oxidized guanine base G(N1-H)• (Scheme 4) [126], which is identical to the radical produced via a radiation-mediated direct ionization process [13,93,117,118,119,120,121,122,123,124] as well as via radiation-produced •OH addition to the C4=C5 bond of a guanine base followed by water elimination from the adduct radical [13,93].

We have extended our work to various pyrimidine nucleosides with azido modification at the C5-site of the pyrimidine base; e.g., C5-azidomethyl-2′-deoxyuridine (5-AZmdU; and our results show that radiation-produced electrons can be converted to the highly damaging RNH• at 77 K [128]. Based on these results, we hypothesize that site-specific incorporation of azido-modified nucleosides into genomic DNA would augment radiation-induced damage in cells owing to the reactions of aminyl radicals under a reductive environment. Thus, such azido-modified nucleosides show promise as potent radiosensitizers of hypoxic tumor cells.

### 5.3. Evidence of Radiosensitization by Azidonucleosides

A direct correlation between cell death and micronuclei-induction was obtained in HeLa cells exposed at increasing doses of γ-irradiation in the presence of an aqueous solution (0.1 µM) of 3′-AZT [129]. Moreover, 3′-AZT causes significant radiosensitization in irradiated human colon cancer cells [130], in irradiated human larynx squamous carcinoma cells [131], and in irradiated human malignant glioma cells [132]. 3′-AZT substantially enhanced γ-irradiation killing of EBV (Epstein-Barr virus)-transformed lymphoblastoid cells in vitro [133]. In addition, our very recent studies employing a number of C5-azidomethyl and C5-azidovinyl pyrimidine nucleosides show that 5-AZmdU caused significant radiosensitization against EMT-6 breast cancer cells [128]. These results offer an opportunity to investigate the potential use of azido-DNA-models as site-specific radiation sensitizers.

## 6. Conclusions

In summary, this review focused on the various stages of radiation induced formation of electrons and their reactions with DNA systems. The secondary electrons produced, during interaction of radiation with water, continuously lose their energy until thermalized and ultimately fully solvated. The stages are characterized as low energy electrons (LEE), quasi-free electrons (e^−^_qf_), presolvated electrons (e^−^_pre_) and the fully solvated electron (e^−^_aq_). The latest results establish that the simple tetrahedral “cavity model” of the solvated electron satisfactorily reproduces the physical observables, as well as its reaction with DNA bases. LEE are effective in causing strand breaks, and solvated and prehydrated electrons result in base damage that can contribute to the formation of clustered damage sites. The electron is an important entity whose radiation chemistry must be understood in order to comprehend its role in radiation damage in living systems, especially in the area of high LET radiation, such as ion beams. Results cited in this review also point towards a possibly increasing role for radiation-produced electron-mediated radiosensitization by C5-modified pyrimidine nucleosides and azidonucleosides.

## Figures and Tables

**Table 1:** A Comparison of the Cavity Versus Non-Cavity Models of the Solvated Electron in Experiments.

Properties	Experiment	LGS (Non-Cavity)	TB (Cavity)	4-H_2_O (Cavity) ^s^
VDE (eV)	3.3–3.6 ^d^	5.19 ± 0.07 ^r^	2.69 ± 0.01 ^r^	3.45
R_g_ (Å)	2.48 ^e^; 2.35 ^f^	2.46 ± 0.04 ^r^	2.388 ± 0.003 ^r^	2.25–2.5
λ (eV) ^a^	1.728 ^g,h^	1.71 ± 0.06 ^r^	1.940 ± 0.007 ^r^	1.72 ^t^
dE^MAX^/dT (eV/K) ^b^	−2.2 × 10^−3 i^	−5.2 × 10^−3 r^	−(1.9 ± 0.6) ×10^−4 r^	
V (cm^3^/mol) ^c^	+26 ^j^ +26 ± 6 ^u^	−116 ± 27 ^r^	+31 ± 12 ^r^	
Resonance Raman Shift (cm^−1^)	200 ^k^			~200
EPR g-factor shift (ppm)	−1900 ^l^ −1700 ^m^			−1000 to −1350
HFCC (Gauss)	A_iso_ = −0.92 ^n^ B_zz_ = 7.0			A_iso_ = −0.6 B_zz_ = 7.0
Δ_hyd_G* (kcal/mol)	−36.3 ^o^			−37.8 to −38.9
Δ_hyd_H* (kcal/mol)	−30.2 ^o^			−29.9 to −31.7
Δ_hyd_S * (cal/(mol·deg))	20.5 ^o^			26.6–24.0
*E^o^* (V)	−2.95 ^p^ −2.87 ^q^			−2.86

^a^ Optical absorption spectrum peak position. ^b^ Linear temperature dependence of spectral peak position. ^c^ Partial Molar Volume. ^d^ [62,63,64]. ^e^ [35]. ^f^ For water ice at −18 °C [52]. ^g^ [34]. ^h^ [35]. ^i^ [52]. ^j^ [76]. ^k^ Red shift of the O−H stretching frequencies of e^−^_aq_ observed in resonance Raman spectra [53]. ^l^ The EPR g-factor shift of e^−^_aq_. The shift is calculated using (g = g_free_ + ppm/10^6^), where g_free_ is the free electron g-factor (2.0023) [54,55]. ^m^ The ESR g-factor shift of e^−^_aq_ in frozen aqueous solutions [55]. ^n^ ESR isotropic (A_iso_) and anisotropic (B_zz_) hyperfine couplings in low temperature aqueous glasses [57]. ^o^ Thermochemistry at 1 M standard state, see supporting information of reference [32]. Also, see reference [33] for additional experimental values. ^p^ Reduction potential (*E^o^*) of e^−^_aq_ [72]. ^q^ [60,61]. ^r^ [42]. ^s^ [32,72]. ^t^ Unpublished. Calculated for 16H_2_O having tetrahedral cavity using B3LYP-PCM/6−31++G(2df,p). ^u^ [75].

**Table 2:** *E^o^* values of G, A, C, T, and U in Water and in DMF Calculated Using G4 Theory and B3LYP Methods along with the Experimental *E^o^* Values in DMF.

	*E^o^* Versus SHE (V)				
	Theory				
	G4			B3LYP ^b^	Exp. ^a^
	PCM ε = 78		PCM ε = 37	PCM ε = 78	
Base	No water ^i^	Waters (*n*) ^d^	“DMF” ^f^	No water ^i^	DMF
G	−2.84 (−3.31) ^c^	−2.62 (3); −2.66 (2)	−2.86 (ca.−3.0) ^g^	−2.89	< −2.76
A	−2.78 (−2.86) ^c^	−2.57 (3) ^e^	−2.81 (−2.71) ^g^	−2.78	−2.52
C	−2.44 (−2.41) ^c^	−2.31 (2)	−2.48 (−2.56) ^g^	−2.49	−2.35
T	−2.42 (−2.31) ^c^	−2.34 (2)	−2.45 (−2.32) ^g^	−2.37	−2.18
U	−2.33	−2.05 (2)	−2.36	−2.30	−2.07
MUE ^h^	0.24(0.20) V	0.07 V	0.22 (0.20V)	0.16 V	

^a^ Reference [82]. ^b^ Using 6-31++G** basis set. ^c^ Reference [85]. *E^o^* versus SHE was calculated using SHE = 4.36 V and MUE = 0.27 V. ^d^ *n* = number of explicit water molecules. See Figure S3 in the Supporting Information of reference [72]. ^e^ *E^o^* calculated in DMF is −2.54 V and corrected for water (ε = 78.4). ^f^ No explicit DMF molecules were considered. ^g^ Reference [86]. *E^o^* versus SHE calculated in acetonitrile (ε = 36.64) using SHE = 4.44 V. ^h^ MUE (mean unsigned error) average of the unsigned deviations from experiment. For H_2_O experimental values the DMF experimental values were employed with 0.03 V added. ^I^ No explicit water molecules added.
